# Nonspecific stress biomarkers for mortality prediction in older emergency department patients presenting with falls: a prospective multicenter observational study

**DOI:** 10.1007/s11739-024-03693-6

**Published:** 2024-07-03

**Authors:** Lukas Terhalle, Laura Arntz, Felix Hoffmann, Isabelle Arnold, Livia Hafner, Laurentia Picking-Pitasch, Joanna Zuppinger, Karen Delport Lehnen, Jörg Leuppi, Rajan Somasundaram, Christian H. Nickel, Roland Bingisser

**Affiliations:** 1 Emergency Department, University Hospital Basel, University of Basel, Basel, Switzerland; 2 Emergency Department, Campus Benjamin Franklin, Charité - Universitätsmedizin Berlin, Berlin, Germany; 3 Emergency Department, Cantonal Hospital Basel-Landschaft, Liestal, Switzerland; 4 Emergency Department, Cantonal Hospital Basel-Landschaft Campus Bruderholz, Binningen, Switzerland; 5 Medical Faculty, University of Basel and Cantonal Hospital Baselland, Liestal, Switzerland

**Keywords:** Emergency department, Mortality prediction, Biomarkers, D-dimer, Adrenomedullin, Copeptin

## Abstract

**Background:**

Older patients presenting to the emergency department (ED) after falling are increasingly prevalent. Falls are associated with functional decline and death. Biomarkers predicting short-term mortality might facilitate decisions regarding resource allocation and disposition. D-dimer levels are used to rule out thromboembolic disease, while copeptin and adrenomedullin (MR-proADM) may be used as measures of the patient`s stress level. These nonspecific biomarkers were selected as potential predictors for mortality.

**Methods:**

Prospective, international, multicenter, cross-sectional observation was performed in two tertiary and two regional hospitals in Germany and Switzerland. Patients aged 65 years or older presenting to the ED after a fall were enrolled. Demographic data, Activities of Daily Living (ADL), and D-dimers were collected upon presentation. Copeptin and MR-proADM levels were determined from frozen samples. Primary outcome was 30-day mortality; and secondary outcomes were mortality at 90, 180, and 365 days.

**Results:**

Five hundred and seventy-two patients were included. Median age was 83 [IQR 78, 89] years, 236 (67.7%) were female. Mortality overall was 3.1% (30 d), 5.4% (90 d), 7.5% (180 d), and 13.8% (365 d), respectively. Non-survivors were older, had a lower ADL index and higher levels of all three biomarkers. Elevated levels of MR-proADM and D-dimer were associated with higher risk of mortality. MR-proADM and D-dimer showed high sensitivity and low negative likelihood ratio regarding short-term mortality, whereas copeptin did not.

**Conclusion:**

D-dimer and MR-proADM levels might be useful as prognostic markers in older patients presenting to the ED after a fall, by identifying patients at low risk of short-term mortality.

**Trial registration:**

*ClinicalTrials.gov Identifier:* NCT02244983.

**Supplementary Information:**

The online version contains supplementary material available at 10.1007/s11739-024-03693-6.

## Introduction

Falls are frequent in aging populations. Falls in older people present a major public health problem and are a challenge to emergency departments (ED) [1, 2]. Almost one-third of all adults aged 65 years and older report a fall at least once a year [3]. Up to 30% of all ED presentations in this age group are related to falls [1], and they may account for 74.3% of major trauma cases [4]. Falls in older adults seem to be associated with high injury severity, reduced mobility, functional decline, and death [5, 6].

Although the incidence and severity of fall-related complications steadily increase after the age of 60 [3, 4, 6, 7], age is not the only factor with an impact regarding risk of falling and related outcomes. Other factors, such as frailty, gender, and co-morbidity [6], are associated with adverse outcomes among patients presenting to the ED after a fall.

Importantly, ED presentation after a fall may be a warning sign of a range of serious conditions [8]. Older adults, particularly with a history of falls, require a thorough work-up, as the risk of secondary deterioration due to missed diagnoses [9] or delirium [10, 11] is considerable. However, such work-ups may lead to excessive hospitalization in a population at risk for long-term institutionalization [12], bearing the risk of additional morbidity and mortality [13, 14]. Thus, preventing avoidable hospital admissions is important to disposition planning in the ED [15].

To distinguish between patients at risk of short-term mortality and those potentially benefiting from rapid discharge is a multi-dimensional challenge. Age, acuity, complexity, and Activities of Daily Living (ADL) are some important dimensions, but a measure of stress level could be a novel, additional dimension. Stress-related biomarkers as mortality predictors could provide another useful tool for helping with difficult disposition decisions. Other approaches include observation [16], routine clinical chemistry [17], or decision analysis frameworks [18].

Multicenter observation studies on this topic are currently scarce and results vary considerably [19, 20]. As falls are associated with serious conditions [21] and both resource allocation and disposition are a challenge in these patients, there is a need for better risk stratification in older adults presenting with falls. We performed this multicenter study in an undifferentiated population to assess the stress response associated with a fall. We hypothesized that stress levels could be associated with morbidity and mortality—irrespective of the cause of the fall, age, gender, or ADL. Different stress response systems were examined for their predictive power in relation to 30-day mortality in patients admitted to the ED after a fall, using three different biomarkers such as copeptin, D-dimers, and the mid-regional fragment of adrenomedullin (MR-proADM). These biomarkers were selected for easy assessment in the ED, previous data on their prognostic value, and their association with an acute stress response.

## Methods

This report is a secondary analysis of the prospective multicenter observational “Falls of Unknown Origin” (ClinicalTrials.gov Identifier: NCT02244983) study covering four EDs in Northwestern Switzerland and Germany (University Hospital Basel, University Hospital Charité Berlin (tertiary care hospitals), Cantonal Hospital Liestal, Cantonal Hospital Bruderholz (regional hospitals)). Data collection was performed between November 2014 and January 2018.

Patients, aged 65 years and older, who presented to the ED within 24 h after a fall and provided informed consent, were enrolled in the study. Transport accidents and accidents involving machinery were excluded as well as assault and intentional self-harm.

Age, sex, and ADL at presentation [22] were obtained by a designated study team. Blood samples were collected after inclusion and D-dimer levels were immediately determined, extra samples of serum and ethylenediaminetetraacetic acid (EDTA) were frozen at − 80 °C. After the trial’s conclusion, copeptin and MR-proADM were determined in batches from those frozen serum and EDTA samples.

### Assays

A viscosity-based detection method with the STA-R system (Diagnostica Stago S.A.S., Asnières sur Seine, Cedex, France) was used to measure D-dimer levels, with detection rate ranging from 0.27 μg/mL up to 20 μg/mL. Citrate plasma for D-dimer estimation was centrifuged at 3500 rpm for 10 min before measurement.

EDTA plasma and serum samples were defrosted and centrifuged at 3400 rpm for 10 min to prepare the samples for measurement. For MR-proADM and copeptin measurements, automated sandwich chemiluminescence immunoassays on the BRAHMS KRYPTOR system (Thermo Scientific Biomarkers, Hennigsdorf, Germany) were used [23, 24].

The MR-proADM KRYPTOR has a detection range of 0.05–100 nmol/L and a functional assay sensitivity of 0.25 nmol/L.

The copeptin proAVP KRYPTOR has a detection range of 0.7–2000 pmol/L and a functional sensitivity of less than 1.59 pmol/L.

All laboratory personnel performing the assays were masked to the patients’ medical information and the purpose of the study.

We used two different cutoff’s for D-dimer: first, we evaluated a common used cutoff of 0.5 µg/mL; and second, we used an age-adjusted patient-individualized cutoff, which was derived by multiplying age by 10 µg/L, as suggested in patients over 50 years of age [25].

Strictly defined cutoffs for MR-proADM and copeptin do not exist. An arbitrary cutoff for MR-proADM was set at 0.75 nmol/L, which was recommended for safe discharge of patients with nonspecific complaints [26, 27]. For copeptin, we used the cutoff suggested by the assay (13.8 pmol/L).

Previous studies have used the first tertiles as cutoff for prognostication in both copeptin and MR-proADM [24, 26, 27]. Both cutoffs chosen above were comparable to the first tertiles of the respective marker in our population (0.72 nmol/L for MR-proADM and 12.23 pmol/L for copeptin).

### Outcomes

#### Mortality

Mortality was followed-up after 30 days, 90 days, 180 days, and 1 year by questionnaires to the patients’ general practitioners, as well as from data from electronic health records, nursing homes, and hospital discharge reports, including in particular all hospitalized or transferred patients.

#### Gold standard diagnoses

After the 30-day follow-up period, independent outcome assessors reviewed all data obtained from electronic medical records, nursing homes, and hospital discharge reports and questionnaires by the patients’ general practitioners. The outcome assessors were two of four ED physicians with longstanding clinical experience. The outcome assessors were blinded to the study hypothesis. They reviewed all available patient records individually and jointly determined the final gold standard diagnoses. These diagnoses were recorded in written form and as codes according to the International Classification of Diseases and Related Health Problems 10th Revision (ICD-10).

#### Categorizations: injurious fall, non-injurious fall, and acute medical condition

The definition of injurious fall was used as defined by Rohacek [28]:

- Any condition that requires invasive procedures, such as surgery
- Any condition that requires prolonged monitoring, such as acute subdural hematoma
- Any bone fractures

The definition of acute medical condition was used as defined by Rohacek [28]:

- Any condition that requires specific medical therapy, such as antibiotics, diuretics, anticoagulants, or antihypertensive drugs
- Any condition that requires invasive procedures, such as surgery, acute endoscopy, or coronary angiography
- Any condition that requires prolonged monitoring, such as acute stroke, myocardial infarction, respiratory compromise, metabolic disorder, hemodynamic instability, intracranial or gastrointestinal bleeding, anaphylaxis, or suicidal tendency

Two reviewers independently classified all patients according to the following rule: if the definition of an injurious fall was fulfilled, the patient was classified as category 1 (injurious falls). If there was no injurious fall, but a serious medical condition was present according to the definition of Rohacek, the patient was classified as category 2. If no criteria for category 1 or category 2 were fulfilled, the patient was classified as category 3 (non-injurious fall). Differing assessments were discussed and summarized in a joint assessment.

### Statistical methods

Descriptive statistics were expressed as counts and percentages or as medians with interquartile ranges (IQR). Group comparisons were performed using the Mann–Whitney *U* test for continuous and ordinal variables and the Fisher exact test for nominal variables.

Sensitivity, specificity, positive and negative likelihood ratios (LRs) were calculated for each biomarker cutoff as a measure of diagnostic accuracy, with 95% confidence intervals obtained from the Clopper–Pearson interval method. If sensitivity was 100%, a bootstrapping method was used to estimate negative LR [29].

To predict 30-day mortality for each biomarker (D-dimer, copeptin, MR-ProADM), univariate and multivariate logistic regression was performed, and odds ratios (ORs) were reported. Age, gender, and the Katz ADL were included as a covariate in multivariate analysis. Receiver operating characteristic (ROC) plots were constructed, and area under the curves (AUCs) were calculated for all biomarkers. Only cases with all three biomarkers available were used to construct ROC plots and calculate AUCs. Kaplan–Meier survival curves were drawn for illustrative purposes.

Correlation between all variables used in the regression analysis was quantified as Pearson correlation.

We performed completed case analysis, resulting in two different cohorts: cohort 1 contains all patients with both copeptin and MR-proADM levels available, and cohort 2 contains all patients with D-dimer levels measured at presentation.

All statistical analyses were conducted with the R program (Version 4.2.2, R core team, R Foundation for Statistical Computing. Vienna. Austria).

### Ethics

This study was approved by the local ethics committee (identifier 2014-184, ww.eknz.ch) and conducted according to the principles of the Declaration of Helsinki.

## Results

**Fig. 1:**
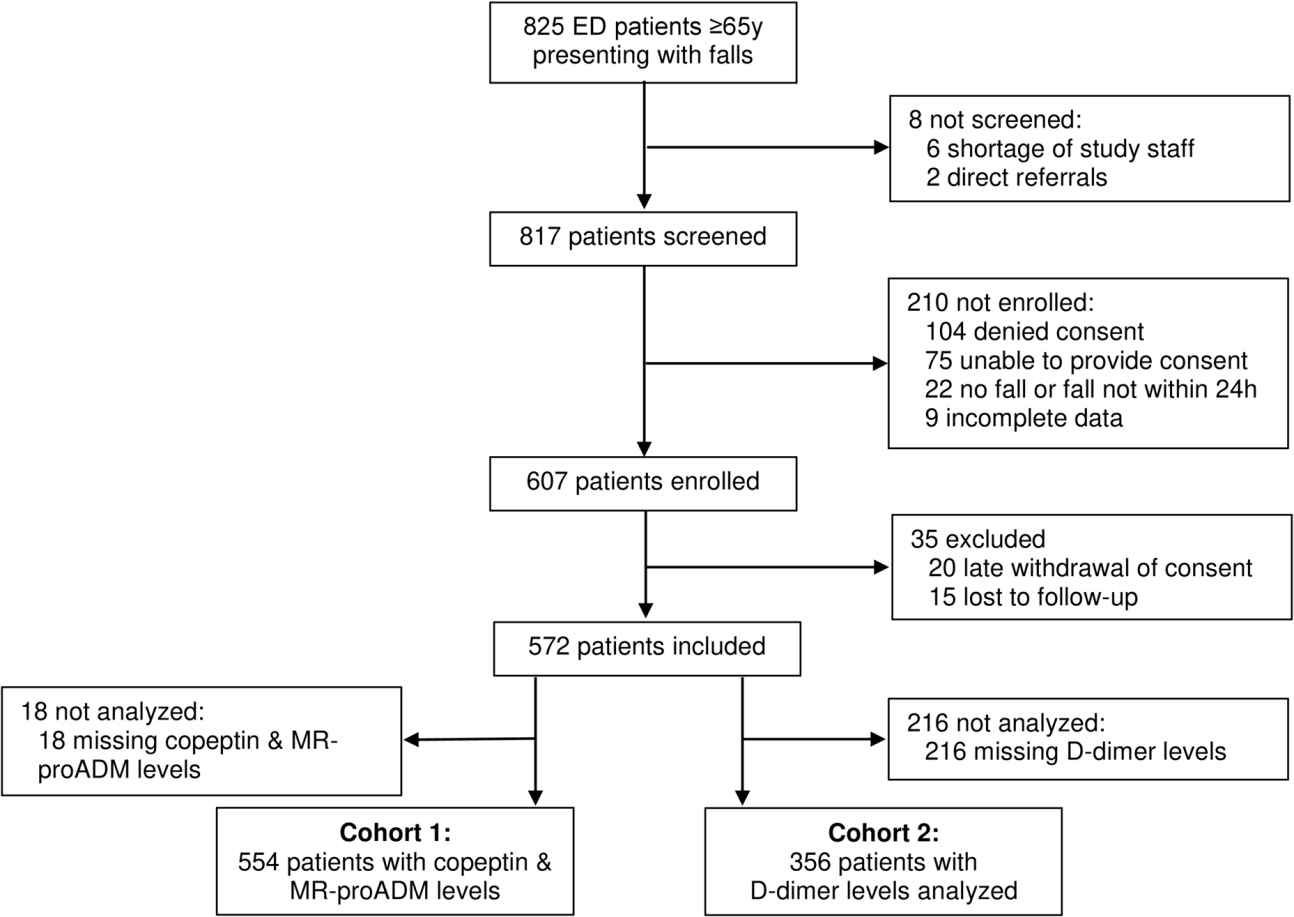
Flow chart of the study population

We included 572 patients with complete data in this analysis (Fig. 1). Median age was 83 years [IQR 77, 89], 67.7% of all patients were female, and 77.4% of patients were admitted as inpatients.

At 30-day follow-up, 18 (3.1%) of all included patients had died. Thirty-one (5.4%) died within 90 days and forty-three (7.5%) within 180 days. At the 365-day follow-up, 77 (13.5%) patients had died, while 495 (86.5%) survived. Details of the 18 patients that died within 30 days are shown in Supplemental Table 3.

Non-survivors were older (median 89 vs. 83 years) and had a lower median ADL index (4 vs. 5) compared to patients who survived 30 days (Table 1).

**Table 1:** Baseline characteristics and biomarker levels for the study population, separated by 30-day mortality

	*n*	All patients	Survivors	Non-survivors	*p* value
Age, *y*	572	83 [77–89]	83 [77–88]	89 [86–92]	0.001
Female gender, *n*	572	387 (67.7%)	372 (67.1%)	15 (83.3%)	0.202
Katz ADL, (0–6)	572	5 [5, 6]	5 [5, 6]	4 [3.25–5]	0.003
Diagnostic category, *n*	570				0.024
Injurious fall		270 (47.4%)	264 (47.8%)	6 (37.5%)	
Acute medical condition		151 (26.5%)	141 (25.5%)	10 (55.6%)	
Non-injurious fall		149 (26.1%)	147 (26.6%)	2 (12.5%)	
Patients admitted, *n*	572	443 (77.4%)	427 (77.1%)	16 (88.9%)	0.389
Copeptin, *pmol/L*	554	19.44 [9.5–45.2]	19.12 [9.2–43.8]	44.98 [23.5–110.4]	0.005
MR-proADM, *nmol/L*	554	0.85 [0.7–1.2]	0.84 [0.7–1.1]	1.50 [1.0–2.6]	< 0.001
D-dimer, *µg/mL*	356	2.14 [1.0–5.7]	2.02 [1.0–5.3]	6.49 [3.4–10.6]	0.002

*for cohort 2: n* = *345 (survivors), n* = *11 (non-survivors)*

Around half (47.4%) of patients were classified as injurious fall, while around a quarter of patients were classified as acute medical condition (26.5%) and as non-injurious fall (26.1%). These rates were comparable compared to patients who survived 30 days (injurious falls: 47.8%, acute medical conditions: 25.5%, non-injurious falls: 26.6%) but differed significantly for non-survivors, where acute medical conditions accounted for the majority (55.6%) of cases, while injurious falls (37.5%) and non-injurious falls (12.5%) accounted for a smaller part.

In 554 patients, MR-proADM as well as copeptin levels were available (cohort 1), whereas D-dimer measurement was available in 356 patients (cohort 2). Three hundred forty-six cases were included in both cohort 1 and cohort 2 (Fig. 1). A comparison of the cohorts regarding baseline characteristics can be found in the Supplemental Table 1.

### Cohort 1

Age and sex distribution as well as overall mortality in cohort 1 were comparable with the complete cohort. Median age was 83.5 years [IQR 77, 89], 67.7% of patients were female and 78% were admitted as inpatients.

At 30-day follow-up, 18 (3.2%) patients had died. Thirty-one (5.6%) died within 90 days and forty-three (7.8%) within 180 days. At the 365-day follow-up, 76 (13.7%) patients had died, while 478 (86.3%) survived. For more details, see Kaplan–Meier curves in Supplemental Figs. 1 and 2.

Based on our predefined cutoffs, 341 (61.6%) patients in cohort 1 had elevated MR-proADM, and 352 (63.5%) had elevated copeptin levels. Median copeptin levels were generally lower in females (17.6 vs. 27.3 pmol/L, *p* =  < 0.001), and there were less females with copeptin levels above the cutoff (57.6% vs. 76%, *p* =  < 0.001), while there was no difference in MR-proADM levels (0.84 vs. 0.9 nmol/L, *p* = 0.23 and 60.5% vs. 63.7%, *p* = 0.51).

Patients with MR-proADM levels above 0.75 nmol/L compared to patients with non-elevated MR-proADM levels were older (85 vs. 80 years, *p* =  < 0.001) and had a higher mortality rate within 30 days (5 vs. 0.5% p = 0.008). Patients with elevated copeptin levels were older (85 vs. 81 years, *p* =  < 0.001) and less likely to be female (61.4 vs. 78.7% *p* =  < 0.001). There was no significantly higher mortality rate within 30 days between the groups with and without copeptin elevation (Supplemental Table 2).

Both, patients with elevated MR-proADM levels, and patients with elevated copeptin levels were more likely to be admitted as inpatients, but neither difference was statistically significant (MR-proADM: 80.6 vs. 73.6%, *p* = 0.059. Copeptin: 80.7 vs. 73.3%, *p* = 0.055).

**Fig. 2:**
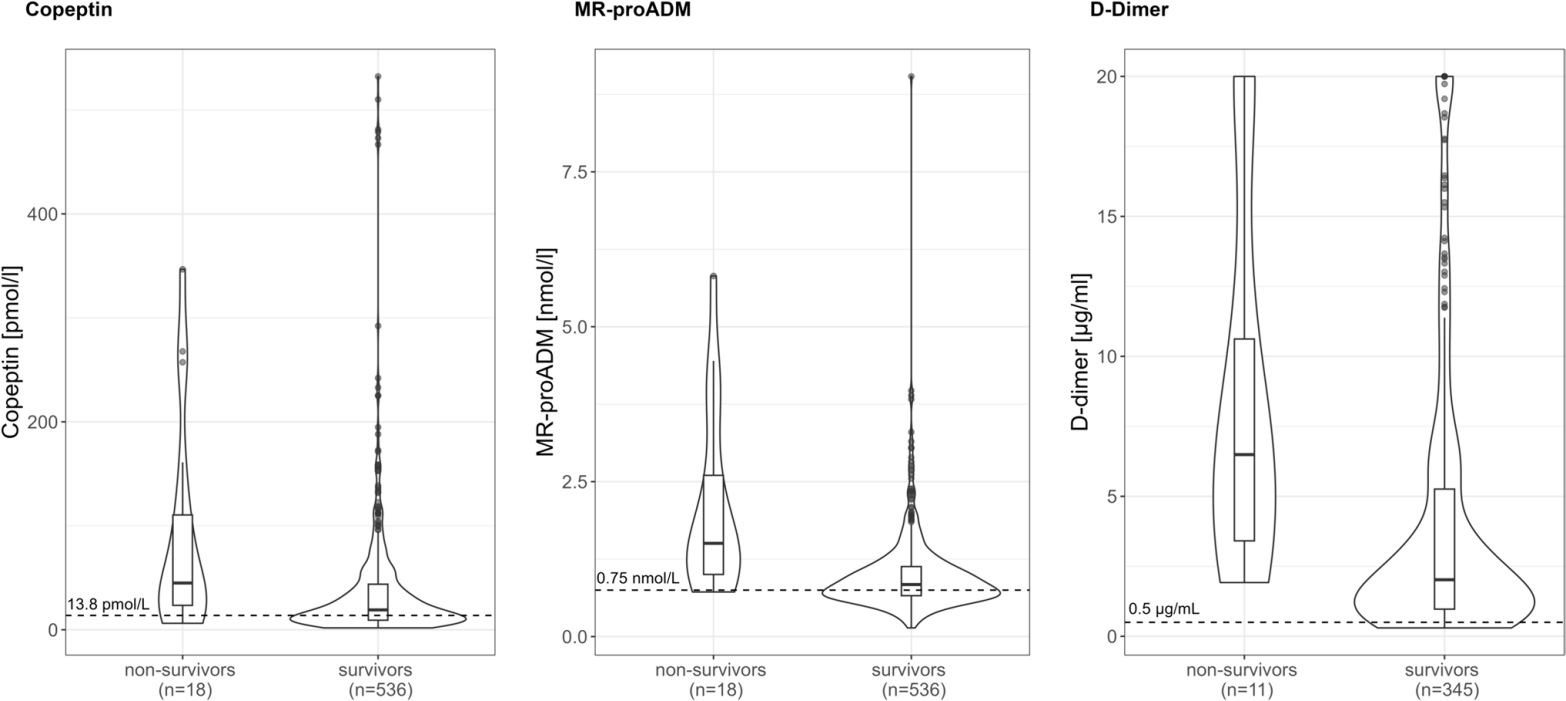
Baseline biomarkers grouped by mortality after 30 days

In cohort 1, the levels of all biomarkers were significantly higher in non-survivors, as illustrated in Table 1, Fig. 2, and Supplemental Table 2.

In cohort 1, 94.4% of patients who died within 30 days and 93.5% of patients who died within 90 days had a MR-proADM level above the predefined cutoff.

Seventy-eight percent of patients who died within 30 days and 74.2% of patients who died within 90 days had a copeptin level above the predefined cutoff.

The predetermined MR-proADM cutoff showed high sensitivity (0.94, 95% CI 0.73–1.0) and low negative likelihood ratio (0.14, 95% CI 0.02–0.95), but low specificity (0.4, 95% CI 0.35–0.44) for mortality within 30 days.

The predetermined copeptin cutoff showed a sensitivity of 0.78 (95%CI: 0.52–0.94), a specificity of 0.37 (95% CI 0.33–0.41), and a negative likelihood ratio of 0.6 (95% CI 0.25–1.44) for mortality within 30 days (Table 2).

**Table 2:** Sensitivity, specificity, and likelihood ratios for mortality within 30 days

	Sensitivity [95% CI]	Specificity [95% CI]	LR + [95% CI]	LR-[95% CI]
Copeptin *cutoff: 13.8 pmol/L*	0.78 [0.52–0.94]	0.37 [0.33–0.41]	1.23 [0.96–1.59]	0.6 [0.25–1.44]
MR-proADM *cutoff: 0.75 nmol/L*	0.94 [0.73–1.0]	0.4 [0.35–0.44]	1.56 [1.37–1.78]	0.14 [0.02–0.95]
D-dimer *cutoff: 0.5 µg/mL*	1.0 [0.72–1.0]	0.10 [0.07–0.14]	1.11 [1.07–1.15]	0.0 [0.0–2.47]
D-dimer *cutoff: age/100 µg/mL*	1.0 [0.72–1.0]	0.22 [0.18–0.26]	1.28 [1.21–1.35]	0.0 [0.0–1.12]

CI: confidence interval, LR + : positive likelihood ratio, LR-: negative likelihood ratio

CI calculated by Clopper–Pearson interval for sensitivity, specificity, LR + of all biomarkers, and for LR- of copeptin and MR-proADM, and by bootstrap for LR- for D-dimers

For elevated MR-pro-ADM levels, the odds ratio of dying within 30 days estimated with univariate logistic regression increased 11.12-fold (95% CI 2.26–201.2) compared to normal levels. After adjusting for age, sex, and ADL level at baseline, the odds ratio was 7.65 (95% CI 1.5–138.94) (Table 3).

**Table 3:** Logistic regression analyses for different elevated biomarkers to predict death within 30 days after presentation

Model	*n*	Univariable OR (95% CI)	Multivariable OR^a^ (95% CI)	AUC^b^
Copeptin > *13.8 pmol/L*	554	2.05 [0.72–7.31], *p* = 0.211	1.00 [1.00–1.01], *p* = 0.329	0.859
MR-proADM > *0.75 nmol/L*	554	11.12 [2.26–201.17], *p* = 0.02	7.65 [1.50–138.94], *p* = 0.051	0.86
D-dimer > = *0.5 µg/mL*	346	2.63 [0.33–340.5], *p* = 0.441	1.20 [0.12–163.39], *p* = 0.904	0.855
D-dimer > = *age/100 µg/mL*	346	6.55 [0.84–844.62], *p* = 0.081	3.08 [0.36–402.40], *p* = 0.368	0.867

^a^Adjusted for age, gender, and ADL index, ^b^AUC, respectively, for multivariable logistic regression

Odds ratio of dying within a year estimated with univariate logistic regression increased 2.63-fold (95%CI 1.15–4.84) for elevated MR-pro-ADM levels. After adjusting for age, sex, and ADL Index at baseline, the odds ratio for mortality was 1.9 (95% CI 1.05–3.55).

For elevated copeptin levels, the odds ratio of dying within 30 days estimated with univariate logistic regression increased 2.05-fold (95% CI 0.72–7.31) compared to normal levels. After adjusting for age, sex and ADL level at baseline the odds ratio for mortality was 1.0 (95% CI 1.0–1.01).

Odds ratio of dying within a year estimated with univariate logistic regression increased 1.38-fold (95% CI 0.83–2.37) for elevated copeptin levels. After adjusting for age, sex, and ADL Index at baseline, the odds ratio for mortality was 0.97 (95% CI 0.56–1.72).

**Fig. 3:**
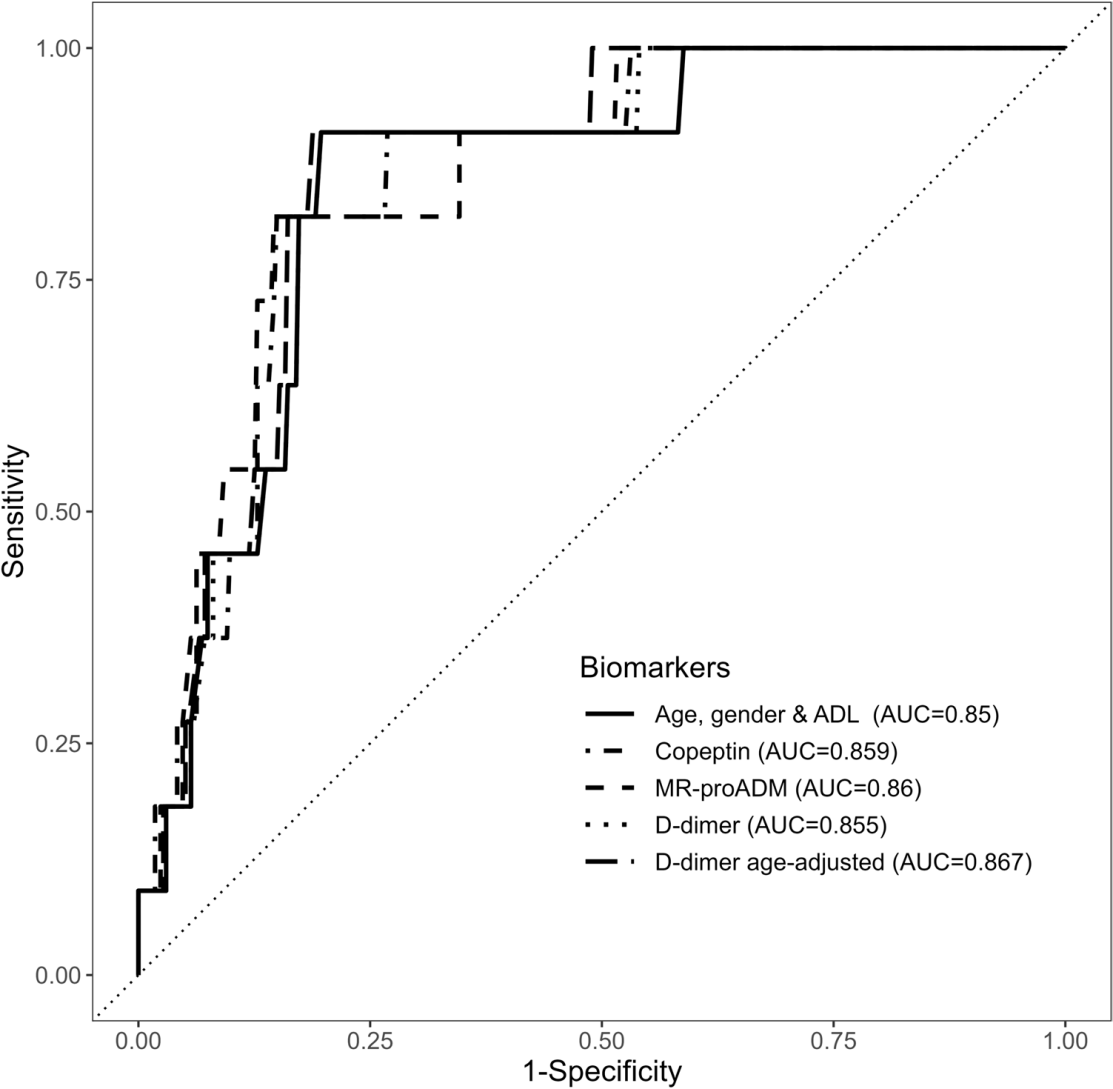
ROC curves for mortality after 30 days predicted by D-dimer, copeptin, and MR-proADM levels, each adjusted for age, gender, and Katz-ADL index against a baseline of these covariates

Adjusting for covariates resulted in change of odds ratio for dying within 30 days for both biomarkers; thus, AUC was only displayed for multivariate logistic regression (Table 3 and Fig. 3).

### Cohort 2

Age and sex distribution as well as overall mortality in cohort 2 were comparable with the complete cohort. Median age was 83 years [IQR 78, 89], 66.3% of patients were female and 78.4% were admitted as inpatients. Eleven (3.1%) patients had died at 30-day follow-up, eighteen (5.1%) died within 90 days, and twenty-five (7%) within 180 days. At the 365-day follow-up, 43 (12.1%) patients had died, while 313 (87.9%) survived. For more details, see Kaplan–Meier curves in Supplemental Figs. 3 and 4.

In cohort 2, there were 321 (90.2%) patients with D-dimer levels above the usual cutoff (< 0.5 µg/mL), and 281 (78.9%) with D-Dimers above the age-adjusted cutoff (patient’s age /100 µg/mL) (Table 1).

Median D-dimer levels were higher in females (2.34 vs. 1.73 µg/mL, *p* = 0.017), and there were more females with D-dimer levels above the age-adjusted cutoff (82.6% vs. 71.7%, *p* = 0.02), but not above the usual cutoff (91.9% vs. 86.7%, *p* =  < 0.132).

Patients with elevated D-dimer levels (cutoff > 0.5) were older (84 vs. 76 years, *p* < 0.001), more likely to be admitted as inpatients (80.1 vs. 62.9%, *p* = 0.029), and had higher mortality rate within 30 days (3.4% vs. 0) compared to patients with non-elevated D-dimer levels (Supplemental Table 2).

In cohort 2, the levels of all three biomarkers were significantly higher in non-survivors, as illustrated in Table 1, Fig. 2, and Supplemental Table 2.

In cohort 2, none of the 11 patients who died within 30 days and the 18 patients who died within 90 days had a D-dimer below the regular, or age-adjusted cutoff.

The regular D-dimer cutoff showed high sensitivity (1.0 95% CI 0.72–1.0) and low negative likelihood ratio (0.0, 95% CI 0.0–2.47), but low specificity (0.10, 95% CI 0.07–0.14). The age-adjusted cutoff shows a higher specificity (0.22, 95% CI 0.18–0.26) without loss of sensitivity (Table 2).

For elevated D-dimer levels, the univariate logistic regression shows an increased odds ratio for mortality within 30 days both for common threshold (OR 2.63 (95% CI 0.33–340.5)) as well as for age-adjusted threshold (OR 6.55 (95% CI 0.84–844.62)). After adjusting for age, sex, and ADL Index at baseline, the odds ratio for mortality within 30 days is 1.2 for common threshold (95% CI 0.12–163.39) and 3.08 for age-adjusted threshold (95% CI 0.36–402.4) (Table 3).

Odds ratio of dying within a year estimated with univariate logistic regression shows an increased odds ratio for elevated D-dimer levels both for common threshold (OR 1.93 (95% CI 0.61–9.71)) as well as for age-adjusted threshold (OR 2.52 (95% CI 1.01–8.02)). After adjusting for age, sex, and ADL Index at baseline, the odds ratio was 1.26 for common threshold (95% CI 0.37–6.54) and 1.98 for age-adjusted threshold (95% CI 0.76–6.46) (Fig. 3).

The correlation between all variables used in the regression analyses is shown in Supplemental Fig. 5.

## Discussion

The main results of our study were the relatively low short-term mortality and the relatively high levels of stress biomarkers in patients presenting to the ED after a fall. Poor prognosis was associated with serious underlying conditions rather than injury pattern.

Patients with low D-dimer and low MR-proADM levels had a low risk of short-term mortality. Although this group was a minority, D-dimers and MR-proADM may be used as “rule-out” tests regarding mortality in patients presenting after a fall. These results are in line with findings in other ED populations with favorable prognoses when biomarker levels were low [26, 30].

The low short-term mortality in patients with negative D-dimer levels was not affected by the use of an age-adjusted cutoff (patient's age/100 µg/mL) [25], but more patients could be ruled out.

Both MR-proADM and D-dimer elevation were shown to be predictive of short-term mortality in our study. Copeptin, which has shown predictive value in previous studies in other populations [31], was outperformed by MR-proADM, as well as by D-dimers, and it correlated with MR-proADM levels.

The rationale for choosing these biomarkers as potential predictors merits discussion:

D-dimers are commonly used to rule out thromboembolic disease, and were elevated in up to 50% in certain ED cohorts [32]. It has been suggested that D-dimers might be used as a non-specific prognostic marker [30, 32, 33], even in a high-risk geriatric population of ED patients presenting with non-specific complaints [34], such as falls of unknown origin. On the other hand, routine assessment of D-dimer levels may be criticized for the possible subsequent search for thromboembolism in case of elevation and the resulting expose to radiation and elevated costs. However, elevated D-dimers should not be taken as an isolated reason for such examinations.

Copeptin, apart from its merits in polyuria-polydipsia syndrome [35], may be used as a measure of a patient’s stress level [31] and can also be used to rule out myocardial infarction in the ED [36].

MR-proADM, as a surrogate for adrenomedullin, rises in response to inflammatory and physiological stress [37].

Copeptin and MR-proADM have both shown to have prognostic value in patients presenting with sepsis, heart disease, lower respiratory tract infections, and chronic obstructive pulmonary disease (COPD) [31, 37–40]. In patients presenting with nonspecific complaints (NSCs), both biomarkers have shown useful prognostic properties [24, 26]. Taken together, the selected biomarkers have been shown to be nonspecific markers of physiologic stress in patients presenting to the ED, and were, therefore, candidates for risk stratification in older patients with falls.

As disposition decisions are difficult in the population studied, stress-marker testing could deliver additional important information and also ameliorate throughput and output of these patients in the ED in times of ED crowding and overload. Although age, acuity, morbidity, and the amount of daily support received are essential considerations for disposition, no current evidence-based framework is convincing. To reduce unnecessary hospital admissions in older patients with nonspecific complaints (a majority accompanied by falls), observation has been suggested [16]. This may lead to more appropriate disposition but depends on the availability of observation units. The reason for observation is mostly indecision due to a lack of a solid prognosis. In unselected ED cohorts, routine clinical chemistry may be used for prediction, but this has never been shown to aid disposition decisions in older patients. Furthermore, there are only a few interventional studies on the use of biomarkers in helping with disposition decisions [27, 41], and in all of them, overruling of algorithms by clinicians was a challenge and a potential bias.

The findings of this study could support ED physicians in identifying patients at low risk of short-term mortality assisting with discharge decisions in these patients. However, mortality is only one preventable outcome, and for older patients, certain outcomes, such as dependency, may be even less desirable [42]. ED physicians tend to take conservative decisions in older patients, as they fear short-term mortality, and identifying low risk patients could potentially reduce unnecessary hospitalizations.

## Limitations

While this study tried to eliminate geographic bias by the inclusion of four different centers in two different countries, the study population predominantly consisted of European natives, limiting the generalizability of the results. Since not all three biomarkers could be examined in all patients of the study population, a possible selection error may have occurred. Because mortality was low for both cohorts, the ORs may be inflated. Patients were only included during day shift, and it is possible that falls at night have more serious consequences. Further, patients in need of life-saving interventions (ESI 1; MTS 1) could not be included due to ethical considerations.

## Conclusion

This study shows that D-dimer and MR-proADM levels could be useful prognostic markers in older patients presenting to the ED after a fall, particularly by identifying patients at low risk of short-term mortality. Copeptin did not provide additional predictive value in this population.

## Supplementary Information

Below is the link to the electronic supplementary material.

Supplementary file 1 (PDF 2823 KB)

## Data Availability

The data analyzed during the presented study are available upon reasonable request from the corresponding author.

## Abbreviations

ED: Emergency department
MR-proADM: Mid-regional proadrenomedullin
ADL: Activities of daily living
IQR: Interquartile range
EDTA: Ethylenediaminetetraacetic acid
OR: Odds ratio
ROC: Receiver operating characteristic
AUC: Area under the curve
COPD: Chronic obstructive pulmonary disease
NSC: Nonspecific complaint
ESI: Emergency severity index
MTS: Manchester triage system
LR +: Positive likelihood ratio
LR-: Negative likelihood ratio
CI: Confidence interval
