# Supplementary material for: Nonspecific stress biomarkers for mortality prediction in older emergency department patients presenting with falls: a prospective multicenter observational study
Source: Intern Emerg Med. 2024 Jul 3;20(2):585–95. doi: 10.1007/s11739-024-03693-6 (PMC11950067; doi:10.1007/s11739-024-03693-6)
Supplement: Supplementary file 1 — (PDF 2823 KB) [file 11739_2024_3693_MOESM1_ESM.pdf]

Supplemental Figure 1

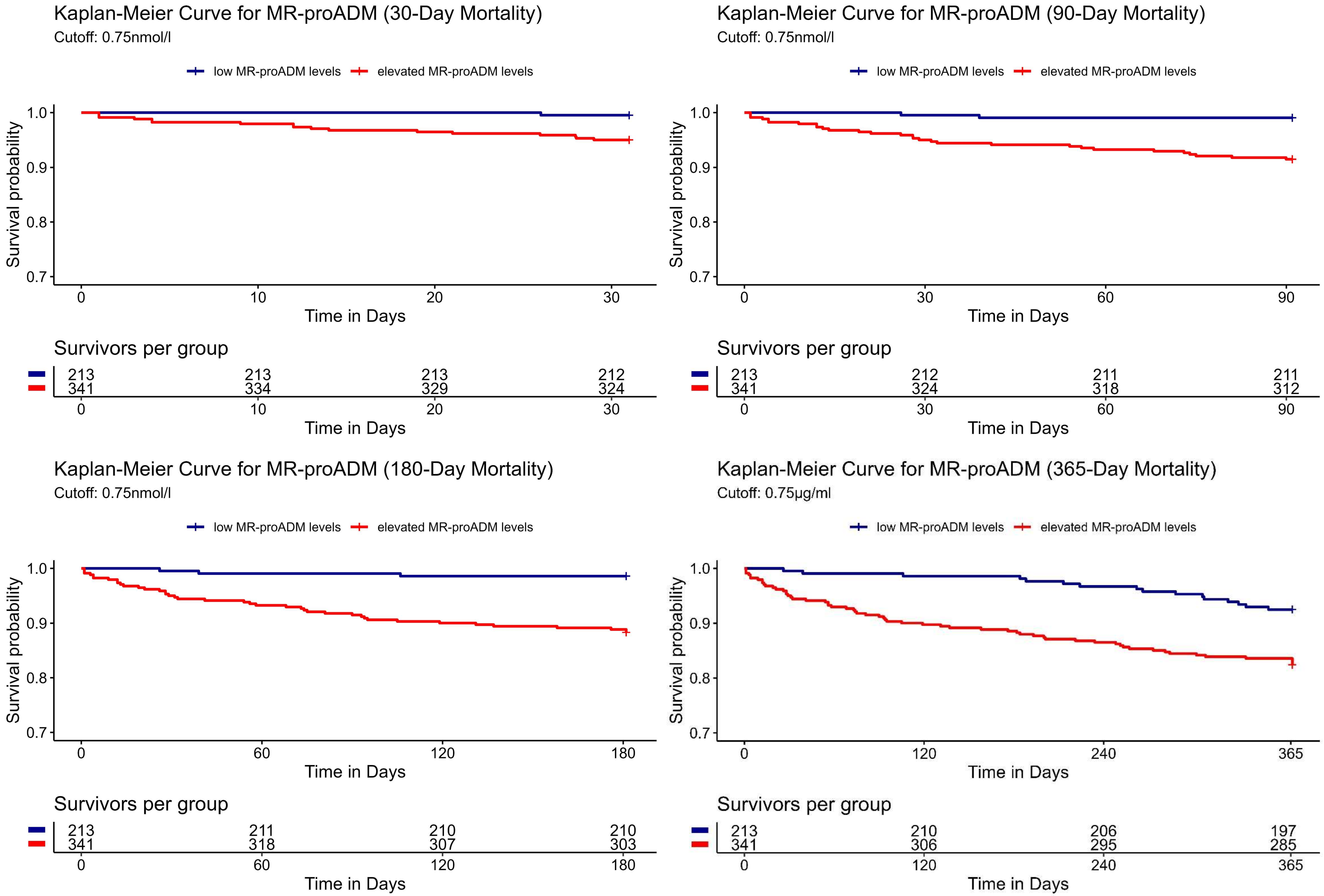

Supplemental Figure 2

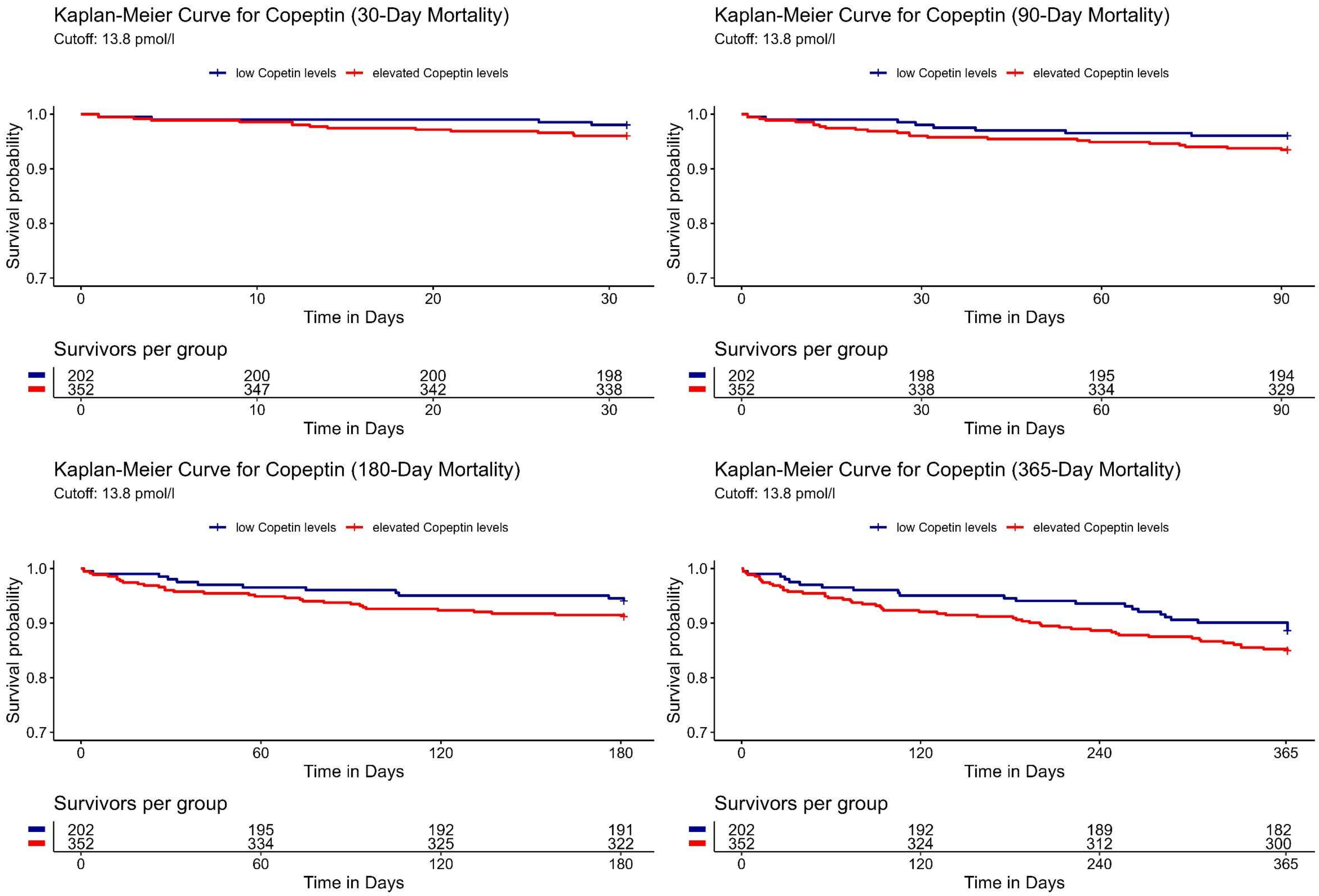

Supplemental Figure 3

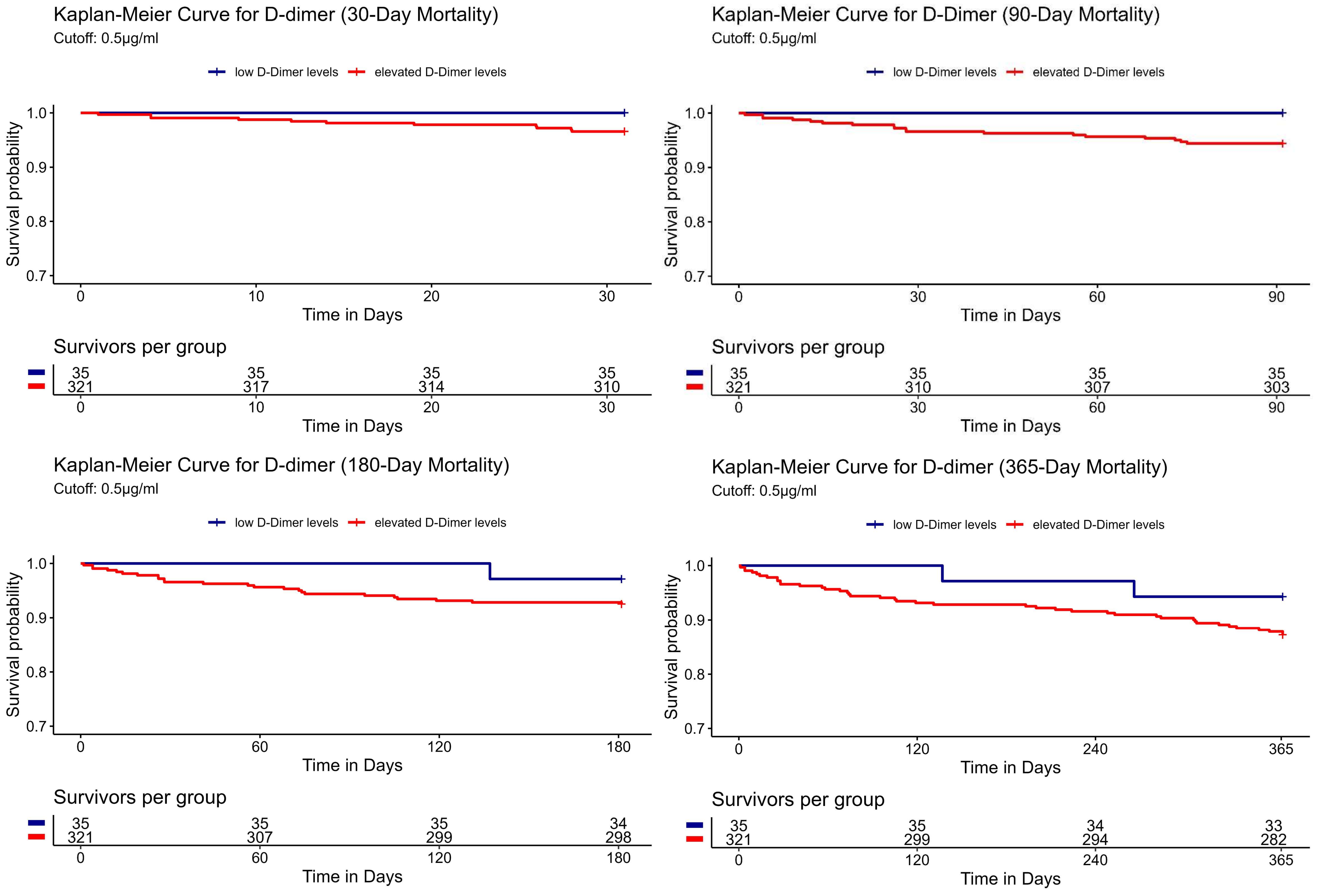

Supplemental Figure 4

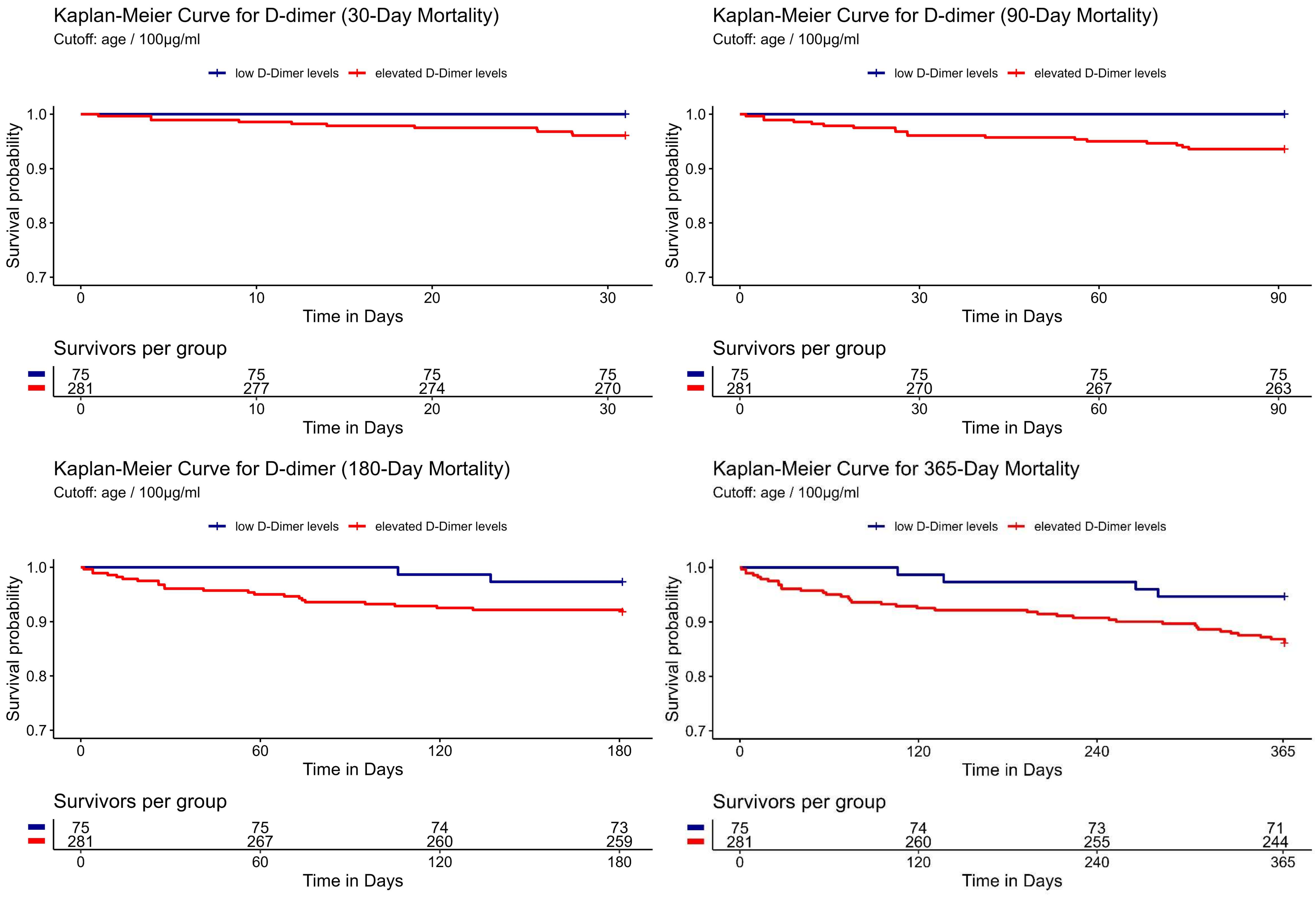

Supplemental Figure 5

Correlation between all data used in logistic regression

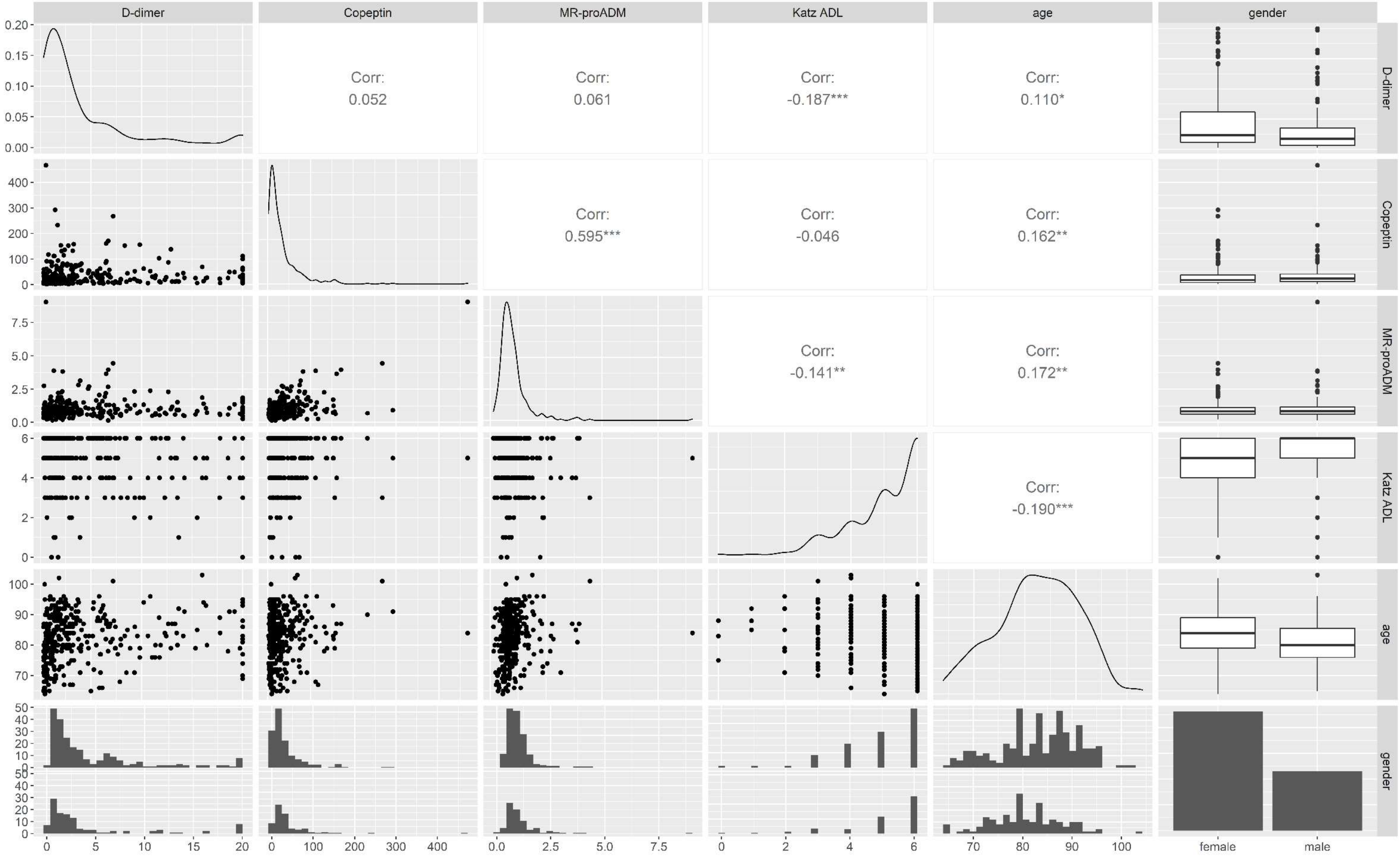

Supplemental Table 1: Comparison of the cohorts

	**Complete Cohort** *n= 572*	**Cohort 1** *n= 554*	**Cohort 2** *n= 356*
Age, *y*	83 [IQR 77, 89]	83.5 [IQR 77, 89]	83 [IQR 78, 89]
Female gender, *n*	387 (67.7%)	375 (67.7%)	236 (66.3%)
Katz ADL, (0-6)	5 [5, 6]	5 [5, 6]	5 [4, 6]
Diagnostic category, *n*	*n = 570*	*n = 552*	*n = 356*
Injurious fall	270 (47.4%)	263 (47.5%)	172 (48.3%)
Acute medical condition	151 (26.5%)	146 (26.4%)	89 (25.0%)
Non-injurious fall	149 (26.1%)	143 (25.8%)	95 (26.7%)
Patients admitted, *n*	443 (77.4%)	432 (78%)	279 (78.4%)
Mortality (30 days), *n*	18 (3.1%)	18 (3.2%)	11 (3.1%)
Mortality (90 days), *n*	31 (5.4%)	31 (5.6%)	18 (5.1%)
Mortality (180 days), *n*	43 (7.5%)	43 (7.8%)	25 (7%)
Mortality (365 days), *n*	77 (13.5%)	76 (13.7%)	43 (12.1%)

Table 1, legend: IQR: Interquartile range

Data are reported as median with IQR and as number with percentage

Supplemental Table 2: Baseline characteristics and Mortality after 30d for the study population, separated by biomarker cutoffs

cutoff: 0.75nmol/L	**Elevated MR-proADM** *n = 341*	**Normal MR-proADM** *n = 213*	**p-value**
Age, *y*	85 [80, 90]	81 [74, 87]	<0.001
Female gender, *n*	227 (66.6%)	148 (69.5%)	0.514
Katz ADL, (0-6)	5 [4, 6]	6 [5, 6]	0.002
Mortality after 30d, *n*	17 (5.0%)	1 (0.5%)	0.008
cutoff: 13.8 pmol/L	**Elevated Copeptin** *n = 352*	**Normal Copeptin** *n = 202*	**p-value**
Age, *y*	85 [79, 90]	81 [76, 87]	<0.001
Female gender, *n*	216 (61.4%)	159 (78.7%)	<0.001
Katz ADL, (0-6)	5 [4, 6]	6 [5, 6]	0.005
Mortality after 30d, *n*	14 (4.0%)	4 (2.0%)	0.304
cutoff: 0.5 µg/mL	**Elevated D-Dimer** *n = 321*	**Normal D-Dimer** *n = 35*	**p-value**
Age, *y*	84 [79, 89]	76 [71.5, 80]	<0.001
Female gender, *n*	217 (67.6%)	19 (54.3%)	0.163
Katz ADL, (0-6)	5 [4, 6]	6 [5, 6]	0.901
Mortality after 30d, *n*	11 (3.4%)	0 (0.0%)	0.61
cutoff: age / 100 µg/mL	**Elevated D-Dimer** *n = 281*	**Normal D-Dimer** *n = 75*	**p-value**
Age, *y*	84 [79, 89]	79 [72, 85]	<0.001
Female gender, *n*	195 (69.4%)	41 (54.7%)	0.024
Katz ADL, (0-6)	5 [4, 6]	6 [5, 6]	0.053
Mortality after 30d, *n*	11 (3.9%)	0 (0.0%)	0.13

Supplemental Table 1, legend: IQR: Interquartile range,

Data are reported as median with IQR and as number with percentage

Supplemental Table 3: *Details of the 18 patients who died within 30 days*

Patient	Presenting complaints other than fall	Outcome Assessor Diagnoses (ICD-10 codes)	Cause of death	Autopsy
89 y/o female	Back pain, chest pain, dizziness, depressed mood	K56.4 Fecal impactation<br>K56.7 Ileus	Ileus	Unknown*
94 y /o female	Gait disturbance, weakness/fatigue, hip pain, confusion	N19 Renal failure	Renal failure	Unknown*
95 y /o female	Weakness/fatigue, hip pain	S32.5 Fracture of os pubis<br>D50.0, Iron deficiency anemia	Readmission: Hypovolemic shock with upper gastrointestinal bleeding	Yes
88 y /o female	Hip pain, confusion	S70.0 Contusion of hip<br>F03 Unspecified dementia	Progressive frailty syndrome, possible stroke	No
84 y /o female	Weakness/fatigue, leg pain	S82.8 Trimalleolar fracture<br>R54 Senility<br>F03 Unspecified dementia	Postoperative heart failure	No
87 y /o female	Gait disturbance, weakness/fatigue, back pain, dyspnea, nausea, emesis, anorexia, dizziness	J96.0 Acute respiratory failure<br>R54 Senility<br>S00.9 Superficial injury of head	Acute respiratory failure caused by pneumonia and heart failure	No
101 y /o female	Gait disturbance, weakness/fatigue, leg pain, headache, dyspnea, anxiety, confusion, paresthesia, nausea	R 57.9 Shock of unclear cause,<br>I50.19 cardiac decompensation due to atrial fibrillation<br>S 70.0 contusion of hip	Not known, died 1 day after discharge	No

89 y /o female	Gait disturbance, weakness/fatigue, hip pain, dyspnea, anxiety, confusion, palpitations	A09.9 Gastroenteritis and colitis of unspecified origin<br>G40.8 Other epilepsy<br>F03 Unspecified dementia	Diverticulitis, Clostridium colitis, sepsis during hospitalization, palliative care	No
86 y /o female	Gait disturbance, weakness/fatigue, dyspnea, nausea, emesis, anxiety, anorexia, paresthesia,	R54 Frailty syndrome<br>I70.0 Atherosclerosis of aorta	Sepsis, heart failure	Unknown*
87 y /o female	Gait disturbance, weakness/fatigue, nausea	C78.7 Colonic cancer with hepatic metastases<br>R54 frailty	Secondary fall resulting in death at home. Palliative care	Unknown*
90 y /o female	Weakness/fatigue, arthralgia, dyspnea, anorexia,	I50.1 Chronic heart failure,<br>R54 Frailty syndrome	Heart failure, palliative care	Unknown*
92 y /o female	Gait disturbance, weakness/fatigue, dyspnea, nausea, anorexia, palpitation	S06.5 Traumatic subdural haemorrhage<br>K57.3 Diverticular disease of large intestine without perforation or abscess	Complications of diverticulitis (perforation and bleeding), discharged for patient preferences	No
92 y /o male	Gait disturbance, weakness/fatigue, confusion, paresthesia, dyspnea, anorexia	N39.0 Urinary tract infection<br>J90 Pleural effusion, hematothorax<br>S00.9 Superficial injury of head	Intraoperative shock during empyema evacuation. Empyema development during hospitalization	Yes
67 y /o male	Gait disturbance, dizziness	E87.1 Hypoosmolar hyponatremia<br>R26.8 Other and unspecified abnormalities of gait and mobility<br>S01.9 Open wound of head	Sudden cardiac arrest 4 weeks after discharge, an echocardiogram 3 days prior to death showed normal left ventricular function, no regional wall abnormalities	No
81 y /o female	Gait disturbance, weakness/fatigue, hip pain, dyspnea, palpitation, dizziness	J18.0 Bronchopneumonia<br>I50.1 Left ventricular failure<br>S72.0 Fracture of neck of femur	Postoperative pulmonary embolism	Yes

87 y /o male	Gait disturbance, weakness/fatigue, arthralgia, hip pain, dyspnea, paresthesia, anorexia	J18.9 Pneumonia<br>S72.1 Pertrochanteric fracture<br>G62.9 Polyneuropathy	Septic shock	Unknown*
93 y /o female	Gait disturbance, weakness/fatigue, anorexia	S72.4 Fracture of distal femur<br>R54 frailty<br>F03 Unspecified dementia	Postoperative pneumonia, left ventricular failure secondary to atrial fibrillation with tachycardia	No
96 y /o female	Gait disturbance, dyspnea, palpitation	S72.1 Pertrochanteric fracture<br>R54 frailty	Acute respiratory deterioration, suspected postoperative pulmonary embolism	No

Abbreviations: ICD-10, International Statistical Classification of Diseases and Related Health Problems 10th Revision

* Information on autopsy is only available for patients hospitalized in University Hospital Basel or Hospital Liestal
